# Supplementary material for: Proposed Standard Test Protocols and Outcome Measures for Quantitative Comparison of Emissions from Electronic Nicotine Delivery Systems
Source: Int J Environ Res Public Health. 2022 Feb 14;19(4):2144. doi: 10.3390/ijerph19042144 (PMC8871820; doi:10.3390/ijerph19042144)
Supplement: Supplementary file 1 [file ijerph-19-02144-s001.zip › Supplementary Materials/Supplementary Material 1.pdf]

Supplemental Slides to accompany the article:

Proposed Standard Test Protocols and Outcome Measures for Quantitative Comparison of Emissions from Electronic Nicotine Delivery Systems. DOI...

Edward C. Hensel, Nathan C. Eddingsaas, Qutaiba M. Saleh, Shehan Jayasekera, S. Emma Sarles, A. Gary DiFrancesco, and Risa J. Robinson

Funding: Research reported in this publication was supported by the National Institute Of Environmental Health Sciences (NIEHS) of the National Institutes of Health and FDA Center for Tobacco Products (CTP) under Award Number R21ES029984. The content is solely the responsibility of the authors and does not necessarily represent the official views of the NIH or the Food and Drug Administration.
Conflicts of Interest: None of the authors have any conflicts of interest to declare.

Study Objectives:

1. Demonstrate an accurate, unbiased Emissions Model (EM) for quantifying aerosol emissions generated from pen- and pod-style ENDS across their respective operating envelope.
2. Identify characteristics of the EM which may be used to compare relative emissions between products.
3. Investigate associations between the EM characteristics and underlying product characteristics.
4. Propose characteristics likely to be effective regulatory parameters to achieve positive outcomes related to public health, reduction in harm potential and clinical guidance.

Thirteen ENDS and ELIQUID combinations were studied.

Product ID Code	Device Manufacturer	Device Model	Consumable Manufacturer	Consumable Labeled Flavor	Unpuffed E-Liquid f_{Nic}	PG Fraction [-]	Mean R_{Coil} [Ω]	Max (1) P_{Nom} [W]	MaxAF [mL/s]	MaxAD [s]
EC07-02	JUUL LABS	Juul	JUUL LABS	Virginia Tobacco	0.052	0.33	1.633	6.7	85	6.5
EC10-01	VUSE	Alto	VUSE	Original	0.052	N/R	1.063	10.2	50	5
EC12-01	SMOK	Novo 2	MAD HATTER JUICE	Classic Tobacco	0.039	0.44	1.463	7.4	58	8
EC14-01	BLU	myblu	BLU	Classic Tobacco	0.02	0.42	1.416	7.7	88	10
EC15-01	NJOY	Ace	NJOY	Classic Tobacco	0.05	0.48	1.034	10.5	58	5.5
EC16-01	UWELL	Caliburn	MAD HATTER JUICE	Classic Tobacco	0.039	0.44	1.405	7.8	88	10
EC17-01	ASPIRE	Breeze 2	MAD HATTER JUICE	Classic Tobacco	0.039	0.44	0.631	17.3	30	10.5
EC18-01	VAPOR4LIFE	V4L Titan	VAPOR4LIFE	Wowbacco	0.023	0.73	2.258	4.8	50	10
EC19-01	LOGIC VAPES	Logic Pro	LOGIC VAPES	Tobacco	0.016	0.77	2.443	4.5	48	12
EC20-01	LOONTECH	Hyde Original	LOONTECH	Spearmint	0.061	N/R	1.61	6.8	86	10
EC22-01	VUSE	Vibe	VUSE	Original Tobacco	0.03	0.23	2.693	4.0	68	6
EC23-01	SMOK	Stick Prince	MAD HATTER JUICE	Classic Tobacco	0.052	0.44	0.174	62.6	90	8
EC24-01	PUFF BAR	Puff Bar	PUFF BAR	Tobacco	0.049	0.54	1.688	6.5	50	3.6

Notes: (1) Power was not experimentally measured in this investigation. Maximum nominal power was, for relative comparison only, computed from $P_{Nom} = V_{Nom}^2 / R_{Coil}$ where V_{Nom} was set to a constant value of 3.3 [VDC].

Results: A graphical summary of the screening emissions model for each ENDS PCU, RESERVOIR, and Consumable.

Emissions Model: EC17-01 ASPIRE Breeze 2 with MAD HATTER JUICE Classic Tobacco

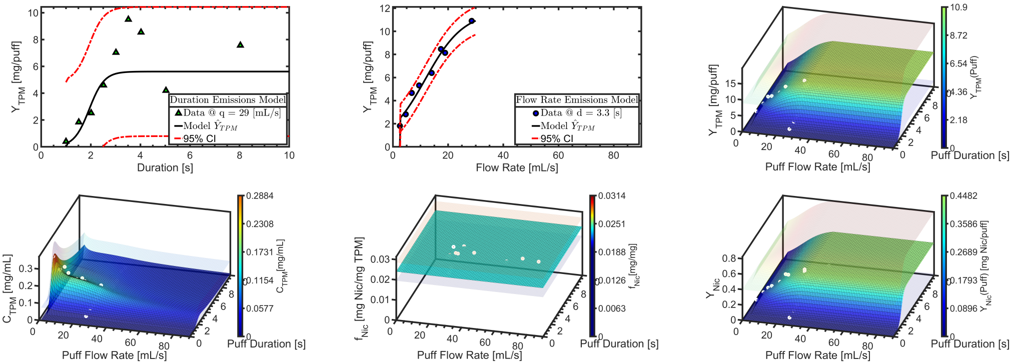

$$\hat{Y}_{TPM}(Puff) = \frac{1}{\min(B_1, B_4)} \left(\frac{B_1}{1+\exp(B_2[q - B_3])} \cdot \frac{B_4}{1+\exp(B_5[d - B_6])} \right); \quad \hat{C}_{TPM} = \frac{\hat{Y}_{TPM}(Puff)}{q \cdot d}; \quad \hat{Y}_{Nic}(Puff) = f_{Nic}(q,d)\hat{Y}_{TPM}(Puff)$$

MinAD = 1.00 s	MinAF = 2.5 mL/s	B_1 = 11.698	B_4 = 5.613	$f_{Nic} = 0.039 \pm 0.019 mg/mg$	Max $Y_{TPM}(puff) = 10.9 \pm 8.97 mg/puff$
MaxAD = 10.5 s	MaxAF = 30. mL/s	B_2 = 0.144	B_5 = 3.301		Max $Y_{Nic}(puff) = 0.425 \pm 0.406 mg/puff$
		B_3 = 11.745	B_6 = 1.940		Max $C_{TPM} = 0.300 \pm 0.072 mg/mL$

Emissions Model: EC14-01 BLU myBlu with BLU Classic Tobacco

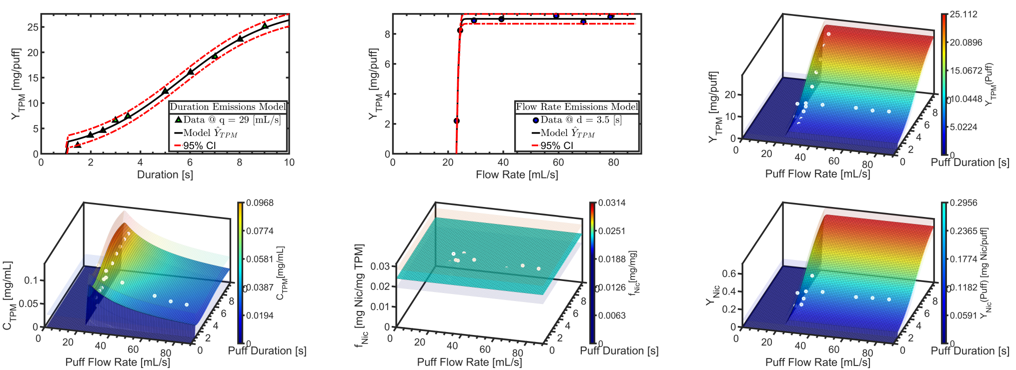

$$\hat{Y}_{TPM}(Puff) = \frac{1}{\min(B_1, B_4)}\left(\frac{B_1}{1+\exp(B_2[q-B_3])}\cdot\frac{B_4}{1+\exp(B_5[d-B_6])}\right);\quad \hat{C}_{TPM} = \frac{\hat{Y}_{TPM}(Puff)}{q\cdot d};\quad \hat{Y}_{Nic}(Puff) = f_{Nic}(q,d)\hat{Y}_{TPM}(Puff)$$

MinAD = 1.00 s
MaxAD = 10.0 s

MinAF = 23. mL/s
MaxAF = 88. mL/s

$B_1 = 9.005$
$B_2 = 2.751$
$B_3 = 23.52$

$B_4 = 28.9$
$B_5 = 0.531$
$B_6 = 5.604$

$f_{Nic} = 0.024 \pm 0.005 mg/mg$

Max $Y_{TPM}(puff) = 23.3 \pm 2.71 mg/puff$
Max $Y_{Nic}(puff) = 0.634 \pm 0.081 mg/puff$
Max $C_{TPM} = 0.110 \pm 0.023 mg/mL$

Emissions Model: EC7-02 JUUL LABS Juul with JUUL LABS Virginia Tobacco

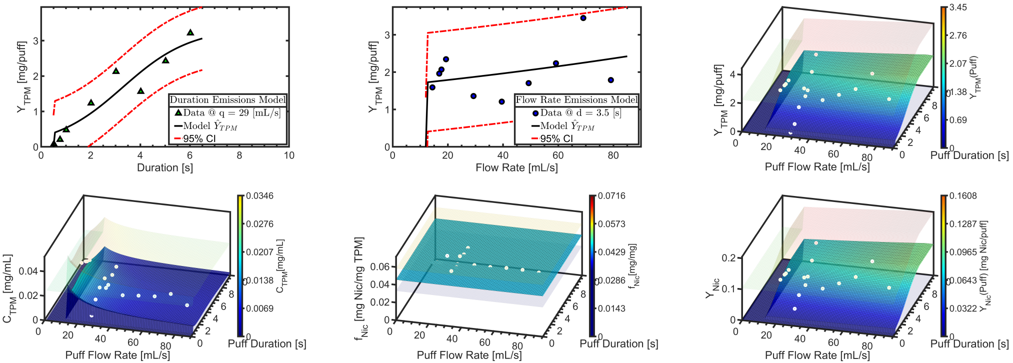

$$\hat{Y}_{TPM}(Puff) = \frac{1}{\min(B_1, B_4)}\left(\frac{B_1}{1+\exp(B_2[q-B_3])}\cdot\frac{B_4}{1+\exp(B_5[d-B_6])}\right); \quad \hat{C}_{TPM} = \frac{\hat{Y}_{TPM}(Puff)}{q\cdot d}; \quad \hat{Y}_{Nic}(Puff) = f_{Nic}(q,d)\hat{Y}_{TPM}(Puff)$$

MinAD = 0.5 s
MaxAD = 6.5 s

MinAF = 12. mL/s
MaxAF = 85. mL/s

$B_1 = 84.69$
$B_2 = 0.0048$
$B_3 = 822.4$

$B_4 = 3.359$
$B_5 = 0.726$
$B_6 = 3.288$

$f_{Nic} = 0.046 \pm 0.012 mg/mg$

Max $Y_{TPM}(puff) = 2.210 \pm 2.25 mg/puff$
Max $Y_{Nic}(puff) = 0.101 \pm 0.104 mg/puff$
Max $C_{TPM} = 0.028 \pm 0.024 mg/mL$

Emissions Model: EC19-01 LOGIC VAPES Logic Pro with LOGIC VAPES Tobacco

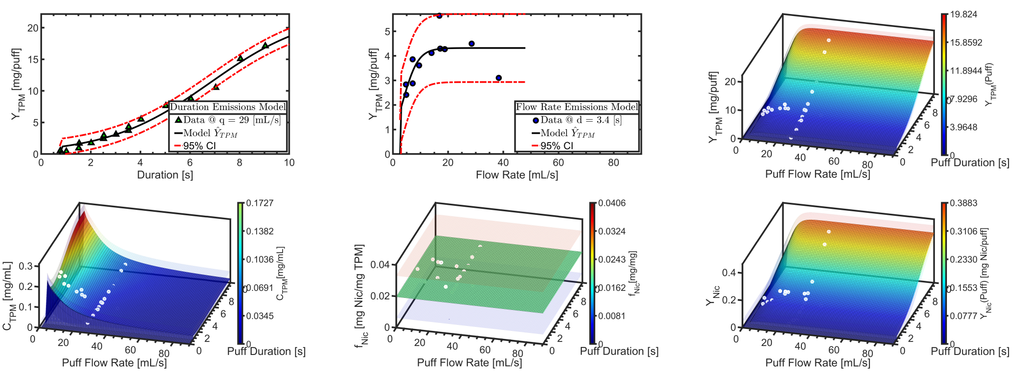

$$\hat{Y}_{TPM}(Puff) = \frac{1}{\min(B_1, B_4)}\left(\frac{B_1}{1+\exp(B_2[q-B_3])}\cdot\frac{B_4}{1+\exp(B_5[d-B_6])}\right); \quad \hat{C}_{TPM} = \frac{\hat{Y}_{TPM}(Puff)}{q\cdot d}; \quad \hat{Y}_{Nic}(Puff) = f_{Nic}(q,d)\,\hat{Y}_{TPM}(Puff)$$

MinAD = 0.75 s
MaxAD = 12. s

MinAF = 2.4 mL/s
MaxAF = 48. mL/s

$B_1 = 4.319$
$B_2 = 0.361$
$B_3 = 3.661$

$B_4 = 22.614$
$B_5 = 0.484$
$B_6 = 6.818$

$f_{Nic} = 0.013 \pm 0.412 mg/mg$

Max $Y_{TPM}(puff) = 20.9 \pm 1.45 mg/puff$
Max $Y_{Nic}(puff) = 0.412 \pm 0.054 mg/puff$
Max $C_{TPM} = 0.276 \pm 0.036 mg/mL$

Emissions Model: EC20-01 LOONTECH Hyde Original with LOONTECH Spearmint

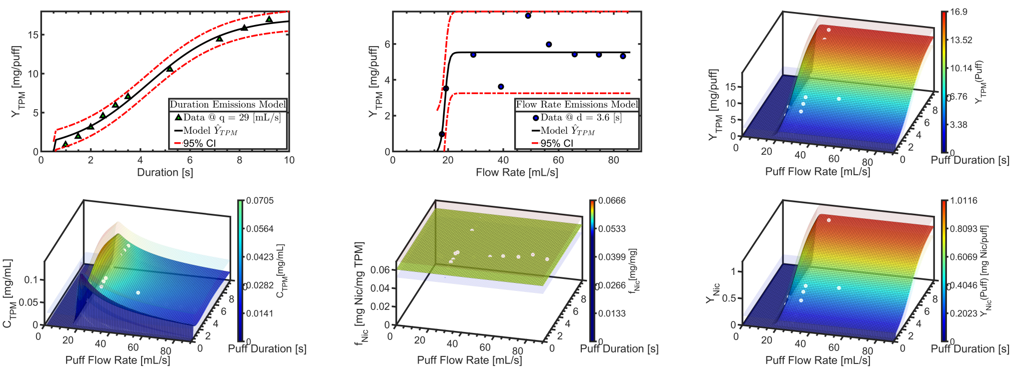

$$\hat{Y}_{TPM}(Puff) = \frac{1}{\min(B_1, B_4)}\left(\frac{B_1}{1+\exp(B_2[q-B_3])}\cdot\frac{B_4}{1+\exp(B_5[d-B_6])}\right); \quad \hat{C}_{TPM} = \frac{\hat{Y}_{TPM}(Puff)}{q\cdot d}; \quad \hat{Y}_{Nic}(Puff) = f_{Nic}(q,d)\hat{Y}_{TPM}(Puff)$$

MinAD = 0.5 s
MaxAD = 10. s

MinAF = 16. mL/s
MaxAF = 86. mL/s

$B_1 = 5.532$, $B_2 = 1.430$, $B_3 = 18.85$

$B_4 = 17.15$, $B_5 = 0.6373$, $B_6 = 4.273$

$f_{Nic} = 0.061 \pm 0.009 mg/mg$

Max $Y_{TPM}(puff) = 16.7 \pm 2.65 mg/puff$
Max $Y_{Nic}(puff) = 0.015 \pm 0.177 mg/puff$
Max $C_{TPM} = 0.114 \pm 0.027 mg/mL$

Emissions Model: EC15-01 NJOY Ace with NJOY Classic Tobacco

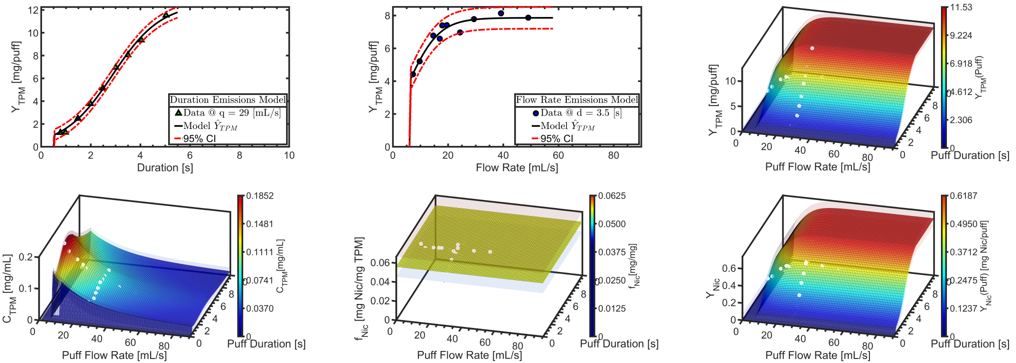

$$\hat{Y}_{TPM}(Puff) = \frac{1}{\min(B_1, B_4)} \left(\frac{B_1}{1 + \exp(B_2[q - B_3])} \cdot \frac{B_4}{1 + \exp(B_5[d - B_6])} \right); \quad \hat{C}_{TPM} = \frac{\hat{Y}_{TPM}(Puff)}{q \cdot d}; \quad \hat{Y}_{Nic}(Puff) = f_{Nic}(q, d)\hat{Y}_{TPM}(Puff)$$

MinAD = 0.5 s
MaxAD = 5.5 s

MinAF = 6. mL/s
MaxAF = 58. mL/s

$B_1 = 7.860$
$B_2 = 0.181$
$B_3 = 5.814$

$B_4 = 12.60$
$B_5 = 1.020$
$B_6 = 2.876$

$f_{Nic} = 0.056 \pm 0.019 mg/mg$

Max $Y_{TPM}(puff) = 11.79 \pm 8.97 mg/puff$
Max $Y_{Nic}(puff) = 0.663 \pm 0.010 mg/puff$
Max $C_{TPM} = 0.190 \pm 0.012 mg/mL$

Emissions Model: EC24-01 PUFF BAR Puff Bar with PUFF BAR Tobacco

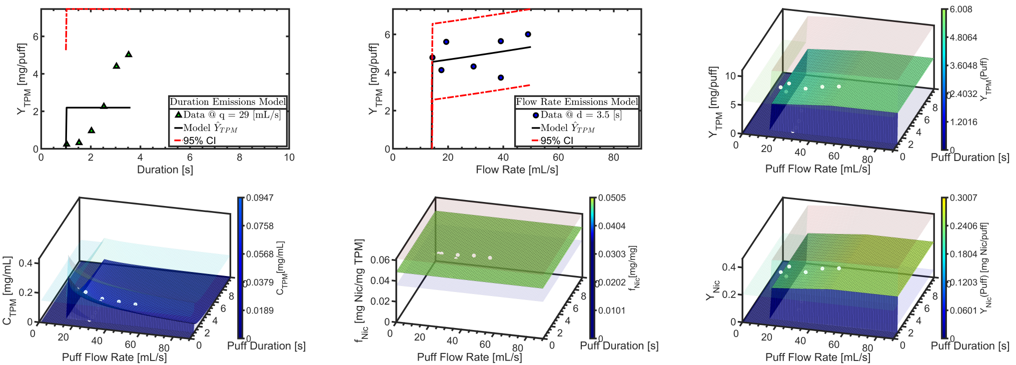

$$\hat{Y}_{TPM}(Puff) = \frac{1}{\min(B_1, B_4)} \left(\frac{B_1}{1 + \exp(B_2[q - B_3])} \cdot \frac{B_4}{1 + \exp(B_5[d - B_6])} \right); \quad \hat{C}_{TPM} = \frac{\hat{Y}_{TPM}(Puff)}{q \cdot d}; \quad \hat{Y}_{Nic}(Puff) = f_{Nic}(q, d)\hat{Y}_{TPM}(Puff)$$

MinAD = 1.0 s
MaxAD = 3.6 s

MinAF = 14.mL/s
MaxAF = 50. mL/s

B_1 =230.1
B_2 = 0.0045
B_3 = 876.1

B_4 = 2.198
B_5 = -5255.4
B_6 = 4.365

$f_{Nic} = 0.049 \pm 0.013 mg/mg$

Max $Y_{TPM}(puff) = 5.343 \pm 5.721 mg/puff$
Max $Y_{Nic}(puff) = 0.260 \pm 0.204 mg/puff$
Max $C_{TPM} = 0.288 \pm 0.144 mg/mL$

Emissions Model: EC12-01 SMOK Novo 2 with MAD HATTER JUICE Classic Tobacco

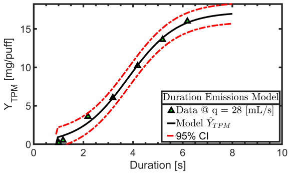

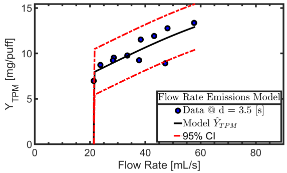

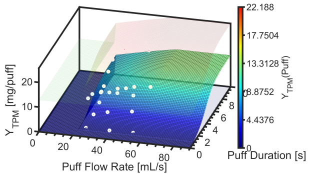

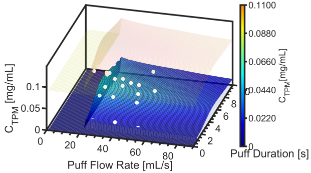

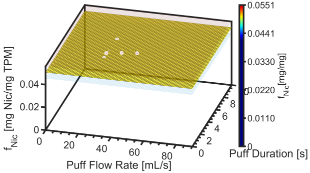

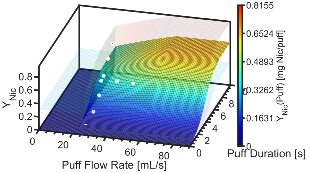

$$\hat{Y}_{TPM}(Puff) = \frac{1}{\min(B_1, B_4)}\left(\frac{B_1}{1+\exp(B_2[q-B_3])}\cdot\frac{B_4}{1+\exp(B_5[d-B_6])}\right); \quad \hat{C}_{TPM} = \frac{\hat{Y}_{TPM}(Puff)}{q \cdot d}; \quad \hat{Y}_{Nic}(Puff) = f_{Nic}(q,d)\hat{Y}_{TPM}(Puff)$$

MinAD = 0.9 s
MaxAD = 8.0 s

MinAF = 21.3 mL/s
MaxAF = 58. mL/s

B_1 =17.113
B_2 = 0.0348
B_3 = 25.603

B_4 = 17.206
B_5 = 1.017
B_6 = 3.798

$f_{Nic} = 0.051 \pm 0.005 mg/mg$

Max $Y_{TPM}(puff) = 12.8 \pm 12.9 mg/puff$
Max $Y_{Nic}(puff) = 0.65 \pm 0.31 mg/puff$
Max $C_{TPM} = 0..057 \pm 0.091 mg/mL$

Emissions Model: EC23-01 SMOK Stick Prince MAD HATTER JUICE Classic Tobacco

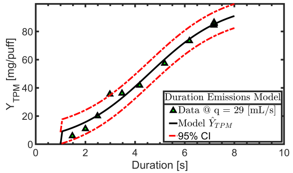

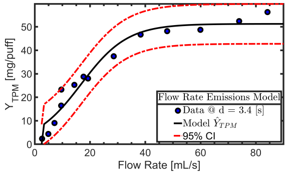

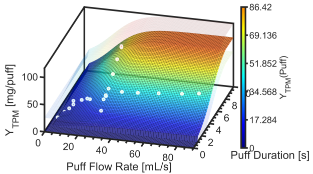

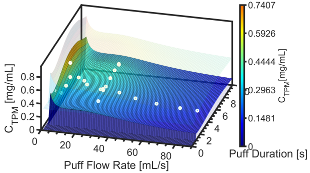

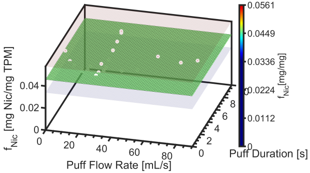

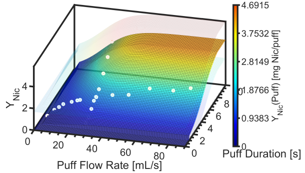

$$\hat{Y}_{TPM}(Puff) = \frac{1}{\min(B_1, B_4)} \left(\frac{B_1}{1+\exp(B_2[q-B_3])} \cdot \frac{B_4}{1+\exp(B_5[d-B_6])} \right); \quad \hat{C}_{TPM} = \frac{\hat{Y}_{TPM}(Puff)}{q \cdot d}; \quad \hat{Y}_{Nic}(Puff) = f_{Nic}(q,d)\hat{Y}_{TPM}(Puff)$$

MinAD = 2.5 s
MaxAD = 8. s

MinAF = 2.5 mL/s
MaxAF = 90. mL/s

$B_1 = 51.253$
$B_2 = 0.120$
$B_3 = 16.803$

$B_4 = 100.66$
$B_5 = 0.654$
$B_6 = 4.581$

$f_{Nic} = 0.046 \pm 0.012 mg/mg$

Max $Y_{TPM}(puff) = 90.94 \pm 25.94 mg/puff$
Max $Y_{Nic}(puff) = 4.175 \pm 1.688 mg/puff$
Max $C_{TPM} = 0.651 \pm 0.312 mg/mL$

Emissions Model: EC16-01 UWELL Caliburn MAD HATTER JUICE Classic Tobacco

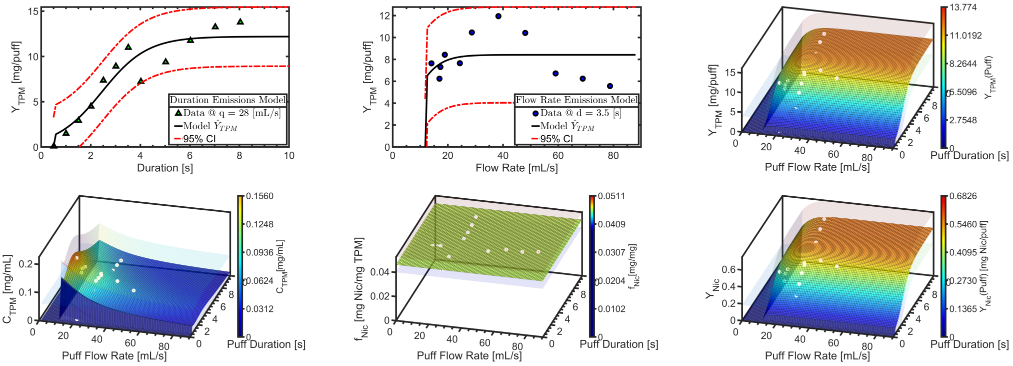

$$\hat{Y}_{TPM}(Puff) = \frac{1}{\min(B_1, B_4)}\left(\frac{B_1}{1+\exp(B_2[q-B_3])}\cdot\frac{B_4}{1+\exp(B_5[d-B_6])}\right);\quad \hat{C}_{TPM} = \frac{\hat{Y}_{TPM}(Puff)}{q\cdot d};\quad \hat{Y}_{Nic}(Puff) = f_{Nic}(q,d)\hat{Y}_{TPM}(Puff)$$

MinAD = 0.5 s
MaxAD = 10. s

MinAF = 11.8 mL/s
MaxAF = 88. mL/s

$B_1 = 8.416$
$B_2 = 0.215$
$B_3 = 6.813$

$B_4 = 12.196$
$B_5 = 1.093$
$B_6 = 2.454$

$f_{Nic} = 0.046 \pm 0.006 mg/mg$

Max $Y_{TPM}(puff) = 12.2 \pm 3.96 mg/puff$
Max $Y_{Nic}(puff) = 0.565 \pm 0.186 mg/puff$
Max $C_{TPM} = 0.167 \pm 0.059 mg/mL$

Emissions Model: EC18-01 VAPOR4LIFE Titan VAPOR4LIFE Wowbacco

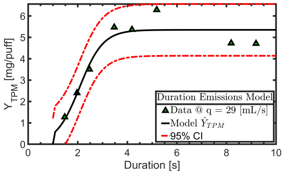

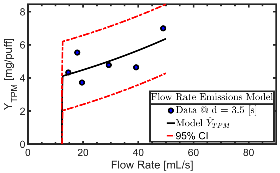

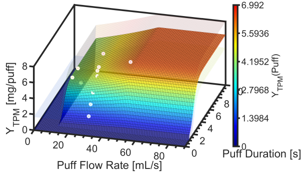

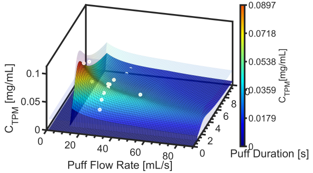

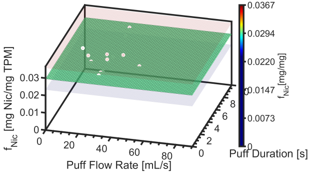

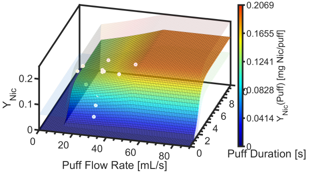

$$\hat{Y}_{TPM}(Puff) = \frac{1}{\min(B_1, B_4)} \left(\frac{B_1}{1 + \exp(B_2[q - B_3])} \cdot \frac{B_4}{1 + \exp(B_5[d - B_6])} \right) ; \quad \hat{C}_{TPM} = \frac{\hat{Y}_{TPM}(Puff)}{q \cdot d} ; \quad \hat{Y}_{Nic}(Puff) = f_{Nic}(q, d)\hat{Y}_{TPM}(Puff)$$

MinAD = 1.00 s
MaxAD = 10. s

MinAF = 12.2 mL/s
MaxAF = 50. mL/s

B_1 = 349.59
B_2 = 0.012
B_3 = 386.3

B_4 = 5.350
B_5 = 2.125
B_6 = 2.081

$f_{Nic} = 0.0296 \pm 0.030 mg/mg$

Max $Y_{TPM}(puff) = 6.365 \pm 1.664 mg/puff$
Max $Y_{Nic}(puff) = 0.188 \pm 0.061 mg/puff$
Max $C_{TPM} = 0.094 \pm 0.019 mg/mL$

Emissions Model: EC10-01 VUSE Alto VUSE Original

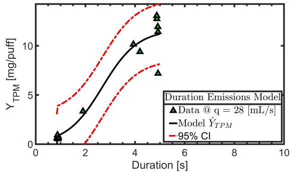

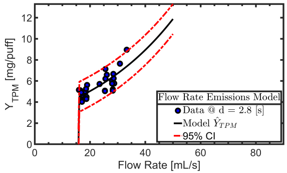

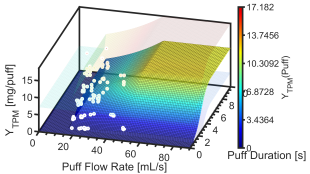

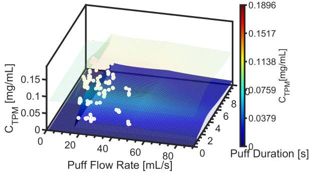

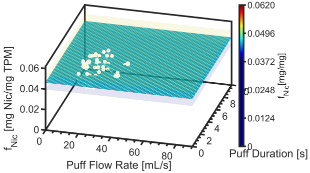

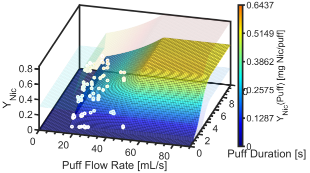

$$\hat{Y}_{TPM}(Puff) = \frac{1}{\min(B_1, B_4)} \left(\frac{B_1}{1 + \exp(B_2[q - B_3])} \cdot \frac{B_4}{1 + \exp(B_5[d - B_6])} \right); \quad \hat{C}_{TPM} = \frac{\hat{Y}_{TPM}(Puff)}{q \cdot d}; \quad \hat{Y}_{Nic}(Puff) = f_{Nic}(q, d)\hat{Y}_{TPM}(Puff)$$

MinAD $= 0.85$ s
MaxAD $= 5.$ s

MinAF $= 15.8$ mL/s
MaxAF $= 50.$ mL/s

$B_1 = 298.59$
$B_2 = 0.029$
$B_3 = 158.37$

$B_4 = 11.636$
$B_5 = 1.404$
$B_6 = 2.686$

$f_{Nic} = 0.046 \pm 0.007 mg/mg$

Max $Y_{TPM}(puff) = 11.43 \pm 7.23 mg/puff$
Max $Y_{Nic}(puff) = 0.531 \pm 0.305 mg/puff$
Max $C_{TPM} = 0.061 \pm 0.085 mg/mL$

Emissions Model: EC22-01 VUSE Vibe VUSE Original

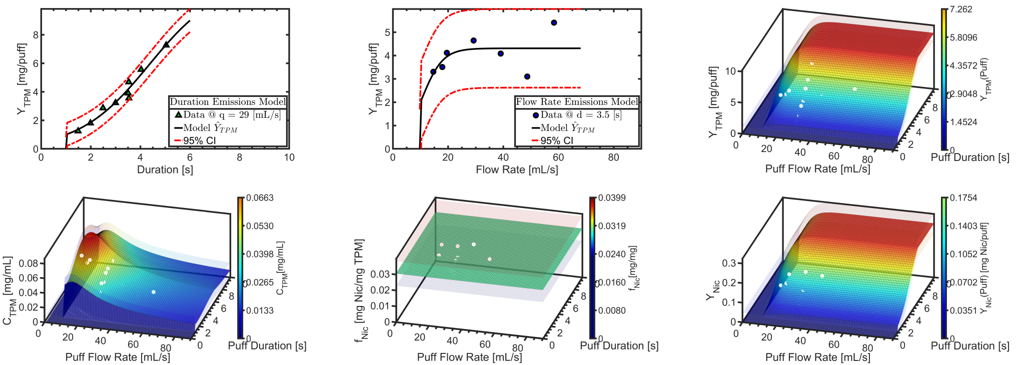

$$\hat{Y}_{TPM}(Puff) = \frac{1}{\min(B_1, B_4)}\left(\frac{B_1}{1+\exp(B_2[q-B_3])}\cdot\frac{B_4}{1+\exp(B_5[d-B_6])}\right); \quad \hat{C}_{TPM} = \frac{\hat{Y}_{TPM}(Puff)}{q\cdot d}; \quad \hat{Y}_{Nic}(Puff) = f_{Nic}(q,d)\hat{Y}_{TPM}(Puff)$$

MinAD = 1. s MinAF = 9.75 mL/s $B_1 = 4.310$ $B_4 = 11.788$ $f_{Nic} = 0.031 \pm 0.008 mg/mg$

MaxAD = 6. s MaxAF = 68. mL/s $B_2 = 0.265$ $B_5 = 0.711$

$B_3 = 10.538$ $B_6 = 4.353$

Max $Y_{TPM}(puff) = 8.998 \pm 1.21 mg/puff$
Max $Y_{Nic}(puff) = 0.276 \pm 0.048 mg/puff$
Max $C_{TPM} = 0.077 \pm 0.010 mg/mL$

Interval Plot: Tobacco Product Maximum Emissions Characteristics Comparison

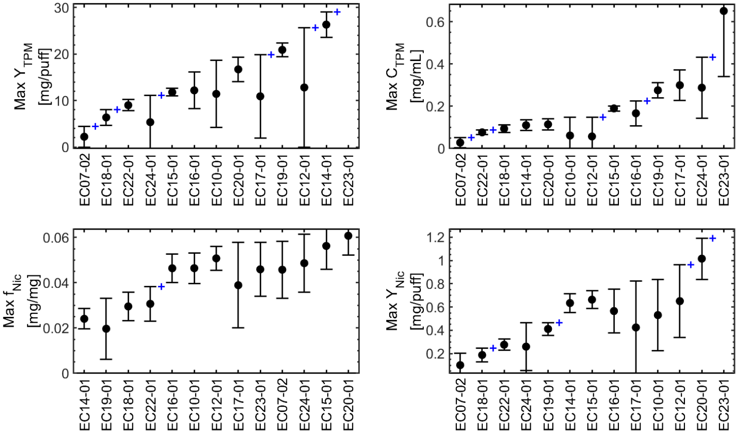

Tobacco Product Emissions Characteristics

Product ID Code	Device Manufacturer	Device Model	Consumable Manufacturer	Consumable Labeled Flavor	Max Y_{TPM} [mg/puff]	Max C_{TPM} [mg/mL]	Max f_{Nic} [mg/mg]	Max Y_{Nic} [mg/puff]
EC07-02	JUUL LABS	Juul	JUUL LABS	Virginia Tobacco	2.210	0.028	0.046	0.101
EC10-01	VUSE	Alto	VUSE	Original	11.433	0.061	0.046	0.531
EC12-01	SMOK	Novo 2	MAD HATTER JUICE	Classic Tobacco	12.814	0.057	0.051	0.650
EC14-01	BLU	myblu	BLU	Classic Tobacco	26.341	0.110	0.024	0.634
EC15-01	NJOY	Ace	NJOY	Classic Tobacco	11.790	0.190	0.056	0.663
EC16-01	UWELL	Caliburn	MAD HATTER JUICE	Classic Tobacco	12.193	0.167	0.046	0.565
EC17-01	ASPIRE	Breeze 2	MAD HATTER JUICE	Classic Tobacco	10.911	0.300	0.039	0.424
EC18-01	VAPOR4LIFE	V4L Titan	VAPOR4LIFE	Wowbacco	6.365	0.094	0.030	0.188
EC19-01	LOGIC VAPES	Logic Pro	LOGIC VAPES	Tobacco	20.911	0.276	0.020	0.412
EC20-01	LOONTECH	Hyde Original	LOONTECH	Spearmint	16.714	0.114	0.061	1.015
EC22-01	VUSE	Vibe	VUSE	Original Tobacco	8.998	0.077	0.031	0.276
EC23-01	SMOK	Stick Prince	MAD HATTER JUICE	Classic Tobacco	90.943	0.651	0.046	4.175
EC24-01	PUFF BAR	Puff Bar	PUFF BAR	Tobacco	5.343	0.288	0.049	0.260

Maximal emissions screening dashboard for consumer-oriented product comparisons.

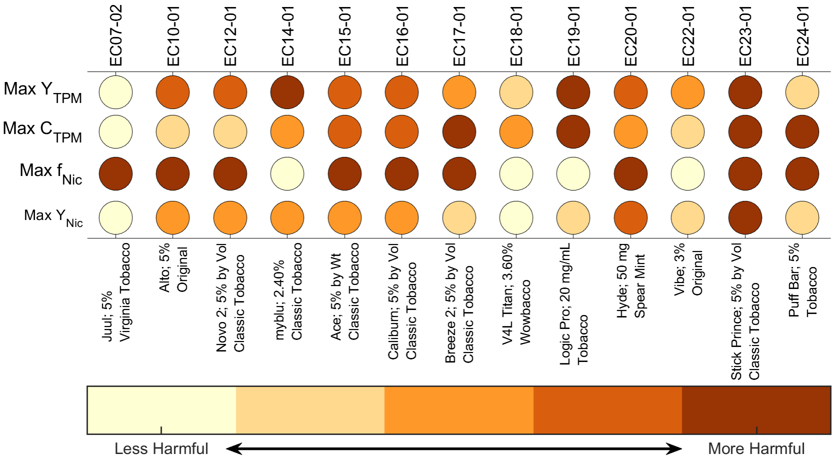

Research Question: Are there associations between Product Emissions Characteristics (what is delivered to the mouth of a user) and Product Design Characteristics (the design, composition and operation of the tobacco product)?

Effect of Product Design Characteristics on Max $\hat{Y}_{TPM}(Puff)$

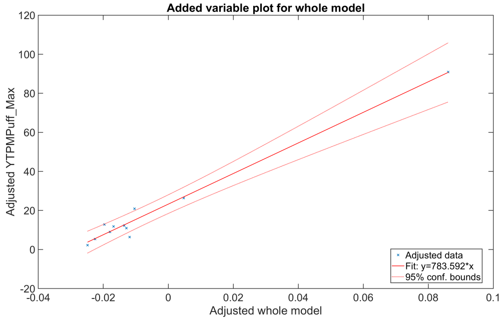

Estimated Coefficients:	Estimate	SE	tStat	pValue
(Intercept)	23.229	28.23	0.8228	0.457
MaxAF	0.2516	0.1078	2.335	0.0798
MaxAD	-1.7727	1.355	-1.3082	0.261
R_{Coil}	-2.5472	5.000	-0.5094	0.637
Max P_{Nom}	1.4806	0.1667	8.882	0.000888
f_{Nic} Unpuffed	-783.4	319.4	-2.453	0.0702
PG Fraction	17.098	15.16	1.128	0.322

Number of observations: 11

Error degrees of freedom: 4

Root Mean Squared Error: 5.62

R-squared: 0.98, Adjusted R-Squared: 0.95

F-statistic vs. constant model: 31.8, p-value = 0.00246

Conclusion: Coil Power is significantly associated ($p < 0.001$) with Max $\hat{Y}_{TPM}(Puff)$.
Conclusion: E-Liquid Nicotine Concentration is marginally associated ($p \approx 0.07$) with Max$\hat{Y}_{TPM}(Puff)$.
Conclusion: Max AF is marginally associated ($p \approx 0.08$) with Max $\hat{Y}_{TPM}(Puff)$.

Effect of Product Design Characteristics on Max $\hat{C}_{TPM}$

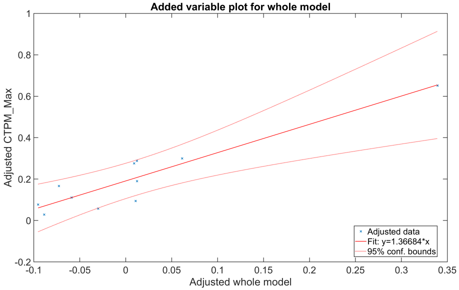

Estimated Coefficients:	Estimate	SE	tStat	pValue
(Intercept)	0.191	0.51052	0.37413	0.7273
MaxAF	-0.0014389	0.0019487	-0.73839	0.50126
MaxAD	-0.01002	0.024504	-0.40893	0.70354
R_{Coil}	-0.012922	0.090422	-0.1429	0.89328
Max P_{Nom}	0.0099909	0.0030143	3.3145	0.02953
f_{Nic} Unpuffed	-1.3392	5.7751	-0.2319	0.828
PG Fraction	0.27256	0.2741	0.99439	0.37632

Number of observations: 11

Error degrees of freedom: 4

Root Mean Squared Error: 0.102

R-squared: 0.868, Adjusted R-Squared: 0.669

F-statistic vs. constant model: 4.37, p-value = 0.0874

Conclusion: Coil Power is significantly associated ($p < 0.03$) with Max $\hat{C}_{TPM}$..

Effect of Product Design Characteristics on Max $\hat{f}_{Nic}(Puff)$

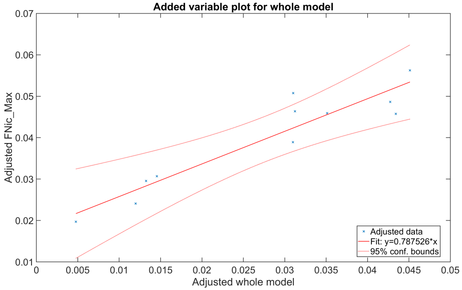

Estimated Coefficients:	Estimate	SE	tStat	pValue
(Intercept)	0.017905	0.032952	0.54335	0.61575
MaxAF	2.2308e-05	0.00012578	0.17736	0.86784
MaxAD	-0.00033789	0.0015816	-0.21363	0.84128
R_{Coil}	-0.0047046	0.0058364	-0.80609	0.46539
Max P_{Nom}	-0.0002499	0.00019456	-1.2844	0.26834
f_{Nic} Unpuffed	0.78746	0.37276	2.1125	0.1022
PG Fraction	0.0087818	0.017692	0.49637	0.64567

Number of observations: 11

Error degrees of freedom: 4

Root Mean Squared Error: 0.00656

R-squared: 0.879, Adjusted R-Squared: 0.698

F-statistic vs. constant model: 4.85, p-value $= 0.0741$

Conclusion: E-Liquid Nicotine Concentration is marginally associated ($p \approx 0.1$) with Max $\hat{f}_{Nic}$.

Effect of Product Design Characteristics on Max $\hat{Y}_{Nic}(Puff)$

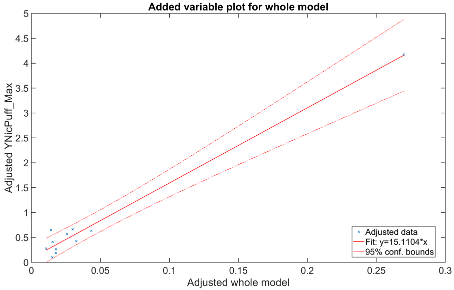

Estimated Coefficients:	Estimate	SE	tStat	pValue
(Intercept)	0.082103	1.3125	0.062552	0.95312
MaxAF	0.0090964	0.00501	1.8157	0.1436
MaxAD	-0.062473	0.063	-0.99164	0.3775
R_{Coil}	-0.02223	0.23248	-0.095622	0.92842
Max P_{Nom}	0.06775	0.0077498	8.7422	0.00094341
f_{Nic} Unpuffed	-15.094	14.848	-1.0166	0.36684
PG Fraction	0.69682	0.70471	0.98881	0.37873

Number of observations: 11

Error degrees of freedom: 4

Root Mean Squared Error: 0.261

R-squared: 0.979, Adjusted R-Squared: 0.948

F-statistic vs. constant model: 31.6, p-value = 0.00249

Conclusion: Coil Power is significantly associated ($p < 0.001$) with Max $\hat{Y}_{Nic}(Puff)$.

Identifying Influential Product Design Characteristics for ENDS Regulation

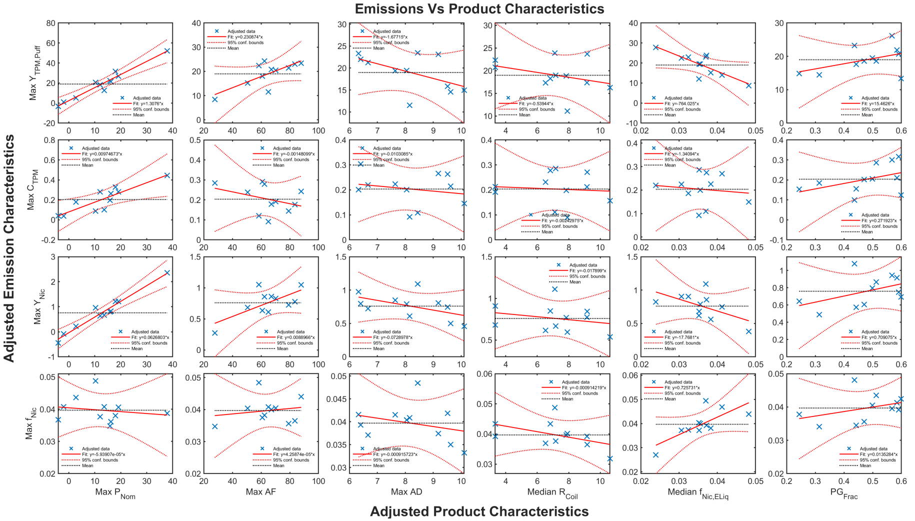

Regulatory Recommendations:

- The product emission characteristics of Max $\hat{Y}_{TPM}$, Max $\hat{C}_{TPM}$, Max $\hat{Y}_{Nic}$ and Max $\hat{Y}_{HPHC}$ should be regulated.
- The product design characteristic of maximum achievable power dissipated in the coil, Max P_{Coil}, should be regulated.

Product User Behavior Characteristics

What is needed: An essential gap

Device Manufacturer	Device Model	Consumable Manufacturer	Consumable Labeled Flavor	Range q_{Puff} [mL/s]	Range d_{Puff} [s]	Range v_{Puff} [mL]	Range N_{PD} [puff/day]	Range N_{PS} [puff/sess]	Range N_{SD} [sess/day]	Session Duration [s]	Session Period [s]
JUUL LABS	Juul	JUUL LABS	Virginia Tobacco								
VUSE	Alto	VUSE	Original								
SMOK	Novo 2	MAD HATTER	Classic Tobacco								
BLU	myblu	BLU	Classic Tobacco								
NJOY	Ace	NJOY	Classic Tobacco								
UWELL	Caliburn	MAD HATTER	Classic Tobacco								
ASPIRE	Breeze 2	MAD HATTER	Classic Tobacco								
VAPOR4LIFE	V4L Titan	VAPOR4LIFE	Wowbacco								
LOGIC VAPES	Logic Pro	LOGIC VAPES	Tobacco								
LOONTECH	Hyde Original	LOONTECH	Spearmint								
VUSE	Vibe	VUSE	Original Tobacco								
SMOK	Stick Prince	MAD HATTER	Classic Tobacco								
PUFF BAR	Puff Bar	PUFF BAR	Tobacco								
